# Dynamical importance of van der Waals saddle and excited potential surface in C(^1^*D*)+D_2_ complex-forming reaction

**DOI:** 10.1038/ncomms14094

**Published:** 2017-01-17

**Authors:** Zhitao Shen, Haitao Ma, Chunfang Zhang, Mingkai Fu, Yanan Wu, Wensheng Bian, Jianwei Cao

**Affiliations:** 1 State Key Laboratory of Molecular Reaction Dynamics, Beijing National Laboratory for Molecular Sciences, Institute of Chemistry, Chinese Academy of Sciences, Beijing 100190, China; 2 School of Chemistry and Chemical Engineering, University of Chinese Academy of Sciences, Beijing 100049, China

## Abstract

Encouraged by recent advances in revealing significant effects of van der Waals wells on reaction dynamics, many people assume that van der Waals wells are inevitable in chemical reactions. Here we find that the weak long-range forces cause van der Waals saddles in the prototypical C(^1^*D*)+D_2_ complex-forming reaction that have very different dynamical effects from van der Waals wells at low collision energies. Accurate quantum dynamics calculations on our highly accurate *ab initio* potential energy surfaces with van der Waals saddles yield cross-sections in close agreement with crossed-beam experiments, whereas the same calculations on an earlier surface with van der Waals wells produce much smaller cross-sections at low energies. Further trajectory calculations reveal that the van der Waals saddle leads to a torsion then sideways insertion reaction mechanism, whereas the well suppresses reactivity. Quantum diffraction oscillations and sharp resonances are also predicted based on our ground- and excited-state potential energy surfaces.

## Abstract (web summary)

It is commonly held that van der Waals wells are inevitable in chemical reactions. Here, the authors show that weak van der Waals forces in the entrance channel of a prototypical complex-forming reaction cause a van der Waals saddle instead, with different dynamical effects from a well at low collision energies.

The van der Waals (vdW) interactions are important in many chemical and biological processes^1^,^2^,^3^ such as the formation of tertiary structures of biopolymers, electron tunnelling in protein crystals and urea's preferential solvation of peptides, to name only a few. The possible role of weak vdW interactions on reaction dynamics is an issue of great interest^2^,^4^,^5^,^6^,^7^,^8^,^9^,^10^,^11^. Although the importance of orientation or stereodynamical effects of the long-range anisotropic interactions was realized earlier^12^,^13^,^14^,^15^,^16^, and has recently become apparent in the emerging field of ultracold bimolecular reactions^17^, it is quite remarkable that the shallow vdW well^2^ in the entrance channel of a classic activated chemical reaction (Cl+H_2_) can have an evident effect on the outcome of the reaction at low collision energies, which has been well recognized since the work of Skouteris *et al*.^2^ Besides the stereodynamical effects on reactivity, the resonances caused by the vdW well are also detected; in particular, it is just found that the shallow reactant and product-valley vdW wells can support quasibound states that give rise to resonances in the F+H_2_ reaction^18^.

However, despite the exciting advances in revealing the effects of the vdW well in direct reactions dominated by the activation barrier^2^,^4^,^5^,^6^,^7^,^8^,^9^,^10^,^11^, the role of the vdW structure in the dynamics of a complex-forming reaction dominated by a deep well is still unclear. Currently, people tend to believe that the vdW wells also exist in other types of chemical reactions. Here we show that the weak vdW forces in the entrance channel of the C(^1^*D*)+D_2_ reaction (Fig. 1a), which is a prototypical complex-forming reaction^19^,^20^,^21^,^22^, give rise to entrance channel vdW saddle (see below for a definition and Supplementary Table 1), which has very different dynamical effects from vdW well at low collision energies. This is achieved with extensive dynamical calculations on the highly accurate *ab initio* potential energy surfaces (PESs) with vdW saddles constructed by us, and our findings are important for acquiring a deeper understanding of complex-forming reactions. For many basic bimolecular reactions such as H+H_2_, Cl+CH_4_ and C+D_2_, the vdW structure results from the dispersion^1^ and quadrupole–quadrupole interactions between the reactants, which are rather weak, and an accurate description on such detailed structures remains a challenge nowadays in the construction of PESs, as is the construction of an excited-state PES.

The C(^1^*D*)+D_2_ reaction is much more difficult to characterize quantum mechanically than the activated reaction type, and has been the subject of many experimental and theoretical works. Experimentally, several kinetic and dynamical studies on the C(^1^*D*)+D_2_ reaction have been reported^23^,^24^,^25^, including a few crossed-beam experiments^24^,^25^. In particular, Liu^24^ has reported the excitation function, and Balucani *et al*.^25^ have obtained the product angular distributions and vibrational branching ratio. Theoretically, Bussery-Honvault *et al*.^26^ have developed a global *ab initio* PES (called Bussery-Honvault-Honvault-Launay (BHL)) for the 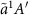 state; later, the *ab initio* data are refitted with the reproducing kernel Hilbert space (RKHS) method to remove some spurious features of the BHL PES, and the modified PES is denoted as the RKHS PES^27^. Although there are other PESs and subsequent dynamical calculations^28^,^29^, the RKHS PES is the most widely used in dynamical studies^27^,^30^,^31^,^32^,^33^,^34^. Most recently, a highly accurate global *ab initio* PES (or Zhang-Ma-Bian-a (ZMB-a)) has been constructed by our group^35^, which is unique in the accurate description of the regions of vdW interactions and around conical intersections (CIs), and further dynamical calculations^36^ performed on this PES for the C(^1^*D*)+H_2_ reaction confirm its accuracy. On the other hand, the 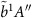 excited-state PES may play an important role, and the only reported global 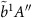 PES is the Bussery-Honvault-Julien-Honvault-Launay (BJHL) surface constructed by Bussery-Honvault *et al*. in 2005 (ref. 37), but the inclusion of the contribution of the BJHL surface worsens the agreement of the theoretical results with the crossed-beam experiments^38^, indicating the deficiency of the BJHL surface.

The main purpose of this work is to study the effects of weak vdW interactions and excited-state PES on a complex-forming reaction. The C(^1^*D*)+D_2_ reaction is chosen, since the crossed-beam scattering data by Liu are available for comparison. To include the contribution of the 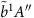 excited state, we have constructed an *ab initio* 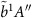 PES. To make it fully convincing, we have performed extensive quantum dynamics (QD) and quasiclassical trajectory (QCT) calculations on the singlet-ground- (both ZMB-a and RKHS) and first-excited-state PESs for the C(^1^*D*)+D_2_ reaction.

## Results

### Excited PES and vdW saddle

**Figure 1:**
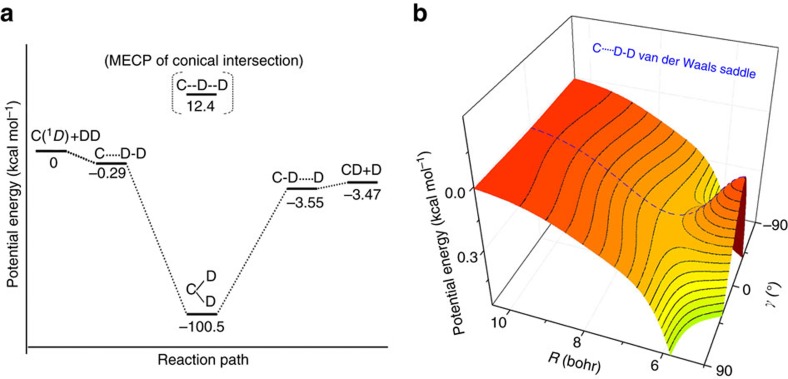
Schematic reaction profile and van der Waals saddle. (**a**) The whole profile along the reaction path. (**b**) A three-dimensional view of the van der Waals saddle in the entrance channel of reaction path is shown where Jacobi coordinates (Supplementary Fig. 1) are used with *r* fixed at D_2_ equilibrium bond length. The ZMB-a potential energy surface for the C(^1^*D*)+D_2_→CD +D reaction is used. The location of the minimum energy crossing point (MECP) on the conical intersection seam between the 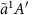 and 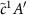 states is indicated.

The construction of accurate global *ab initio* ground-state PESs has played a key role in deepening our understanding of many polyatomic reactions; however, extension to the electronically excited state is rather difficult, and so far much less experience is available. Here a highly accurate global analytical PES for the 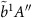 excited state of the title system is constructed by fitting >6,600 symmetry unique *ab initio* energy points (see Methods and Supplementary Methods). The main advantage of our 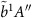 PES is the accurate description of the regions of the vdW interactions and those along the perpendicular insertion and around CIs, and further details are given in Supplementary Discussion. Stationary-point properties on our 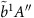 surface are presented in Supplementary Table 2. As can be seen, our 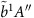 surface has a shallow well at a highly bent *C*_2*v*_ geometry with a depth of 14.4 kcal mol^−1^, which is absent on the BJHL surface^37^, and may play an important role in reaction dynamics. Meanwhile, saddle point instead of minimum is located in the vdW interaction region on our 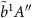 surface, supported by our geometry optimization and frequency analysis with very accurate *ab initio* procedures (Supplementary Table 1). In other words, the present 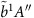 PES constructed by us does not have any vdW well; instead, it has vdW saddles in the entrance and exit channels. This is remarkable, since vdW saddle could be regarded as the counterpart of vdW well, but is a concept unknown before for a chemical reaction. In addition, our *ab initio* calculations (Supplementary Table 1) and further analysis indicate that our ZMB-a surface also has similar vdW saddles. As shown in Fig. 1b, the local potential surface around the vdW stationary point as a function of two variables (C–D–D bending angle and C---DD distance) resembles a saddle very much, and that is why we call it vdW saddle.

Here we further induce a general definition of vdW saddle in a chemical reaction as: in case that the vdW interactions cause a saddle point instead of minimum (vdW well is absent), the PES region surrounding the local saddle point is referred to as vdW saddle. The key property of vdW saddle is its depth, which is here defined as the energy difference between the vdW saddle point and the corresponding asymptote. Then, the depth of the entrance-channel vdW saddle on our 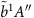 PES is ∼0.22 kcal mol^−1^, somewhat shallower than that on our 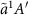 PES (ZMB-a). It is interesting to reveal the dynamical effects of the vdW saddles, and the comparison of dynamical results on the two available PESs (ZMB-a with vdW saddle and RKHS with vdW well) makes it possible, as we will see below.

### Excitation function and reaction mechanism

**Figure 2:**
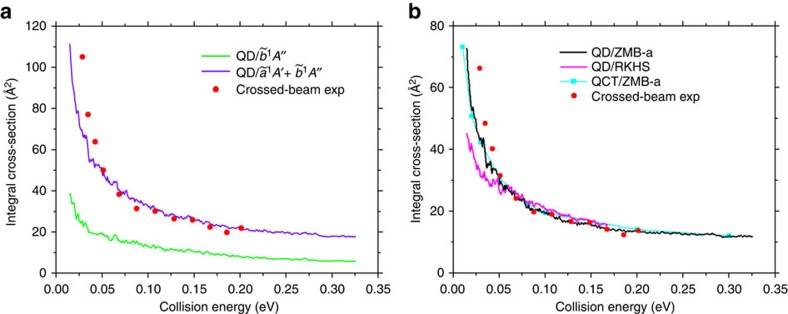
Integral cross-sections as a function of collision energy. (**a**) Accurate quantum dynamics (QD) results calculated on our 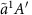 and 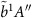 PESs for the C(^1^*D*)+D_2_(*ν*=0, *j*=0) reaction are compared with the crossed-beam experimental data^24^. (**b**) Accurate QD results calculated on our 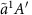 (ZMB-a) and the previous RKHS 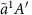 surfaces are compared, along with the crossed-beam experimental^24^ and our quasiclassical trajectory (QCT) results. The relative excitation function is measured in the crossed-beam experiment^24^, which is normalized with the QD value at the collision energy of 0.20 eV.

Figure 2 shows various integral cross-sections (ICSs) calculated by us together with the experimental measurements using the crossed-beam technique by Liu^24^. We see that the ICSs yielded from our QD calculations on our 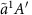 and 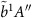 PESs are in very good agreement with experiment. As far as we know, this is the first effort to compare the theoretical results with the crossed-beam relative excitation function by Liu^24^ and the agreement achieved on our surfaces is satisfactory in view of the degree of uncertainty in the measurements. The crossed-beam technique can determine the relative excitation function, but in principle it is unable to determine absolute ICSs^2^. We can also notice from Fig. 2a that the 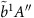 excited PES has an evident contribution to the absolute ICSs, which accounts for >30%. However, as shown in Fig. 2b, the shape of the decreasing ICS as the collision energy rises is quite similar to that obtained on the single ZMB-a surface. On the other hand, it can be easily noticed from Fig. 2b that, although the ICSs calculated on the ZMB-a and RKHS PESs display a similar trend and have close values at higher collision energies, the ICSs from ZMB-a have much larger values at low collision energies (*E*_c_<0.05 eV) and are much more consistent with the crossed-beam excitation function. This phenomenon is attributed to the improvement of our PES (ZMB-a) in the vdW regions in the following analysis. Furthermore, the ICSs calculated with the QCT method on ZMB-a are shown to be in very good agreement with the QD results on the same PES, which justifies the following analysis with the QCT method based on the different topologies of the ZMB-a and RKHS PESs.

The deep well regions of the ZMB-a and RKHS PESs are broadly similar, and both PESs have barriers at similar linear C-D-D geometries and with similar heights. In addition, both PESs support entrance-channel vdW complexes with similar linear equilibrium geometries and energies. However, the two PESs have very different topologies around the entrance-channel stationary points; as mentioned above, the RKHS PES has a vdW well, whereas the ZMB-a PES exhibits a vdW saddle, which is rather flat around the stationary point or local saddle point. It is interesting to mention that the depth of the vdW saddle on ZMB-a is ∼0.3 kcal mol^−1^ (Fig. 1b), which is very close to the depth of the vdW well on RKHS. It should be noted that the *ab initio* calculations for ZMB-a are much larger than those for RKHS, and we think it is important to treat the five states simultaneously (see Methods) due to the mixing of five electronic states in the entrance channel, but we notice that the single-state multireference configuration interaction calculations are performed for the RKHS PES^26^,^27^.

**Figure 3:**
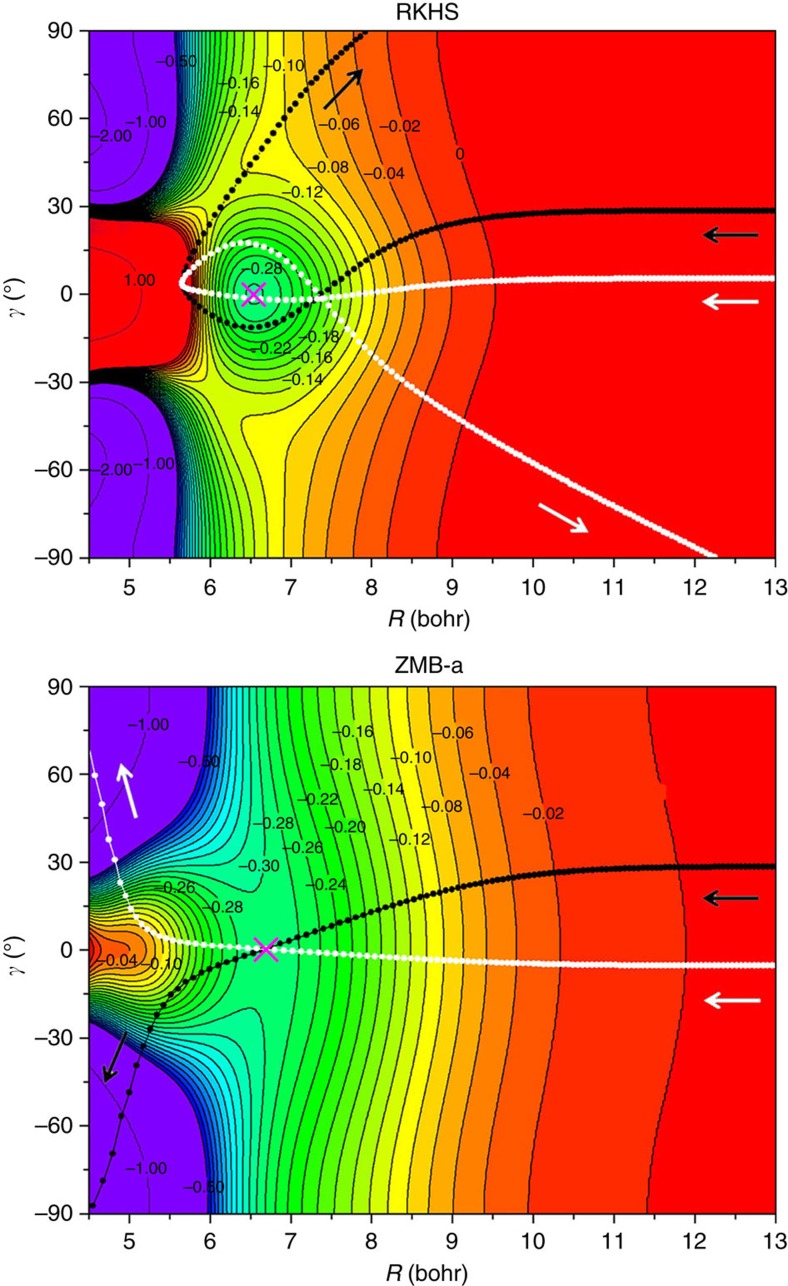
Typical classical trajectories. These are obtained on the RKHS and ZMB-a surfaces with the same initial conditions at the collision energy of 0.005 eV. Jacobi coordinates (Supplementary Fig. 1) are used with *r* fixed at D_2_ equilibrium bond length. (Contours in kcal mol^−1^ relative to the reagent asymptote.) The pink cross is used to indicate the location of the van der Waals minimum on RKHS or van der Waals saddle point on ZMB-a.

To account for the much larger ICSs on ZMB-a than on RKHS at low collision energies, the QCT method is used. The QCT calculations could not only yield dynamical quantities in good accord with accurate QD results for the complex-forming reaction^39^, but also provide an intuitive picture of the reaction mechanism. Reactive classical trajectories leading to the D and CD products on ZMB-a are compared with trajectories from the same initial conditions on RKHS in Fig. 3. We find that most of the trajectories that react to produce CD+D on ZMB-a are turned back by the vdW well in the entrance valley of RKHS before reaching the deep well region, thereby inhibiting the reaction at low collision energies. This kind of effect illustrated in Fig. 3 for two particular classical trajectories is thus considered to be quite general. On the other hand, detailed analyses reveal that, when the C atom approaches a non-rotating D_2_ reagent, the reorientation effects induced by the vdW interactions of ZMB-a lead to a D–D torsion towards the linear C–DD vdW saddle point, and later, the C atom inserts into the D–D bond from a sideways direction. This is illustrated in Supplementary Fig. 2 and called as the D–D torsion then C sideways insertion reaction mechanism here. This mechanism is confirmed by direct examination of trajectories, and the analysis of the relative population of reactive trajectories indicates that it contributes most to the reactivity (Supplementary Fig. 3; Supplementary Discussion) at low collision energies. In addition, Fig. 3 shows that the left part of the vdW well on RKHS rises more steeply as *R* decreases than that of the vdW saddle on ZMB-a, which exhibits a platform structure around *R*=5.0 bohr; we consider this delicate structure on ZMB-a to be realistic since the fitting reproduces our highly accurate *ab initio* calculations very well in this region. Furthermore, we can also see from Fig. 3 that the long-range vdW interactions of ZMB-a extend to longer distances than those of RKHS, thus allowing reactive trajectories with larger impact parameter, which also contributes to the larger ICSs. At higher collision energies where the effects of vdW interactions are evidently weakened, the other small differences between the two surfaces make the ICSs on RKHS even become slightly larger than those on ZMB-a, which further highlights the suppression role of the vdW well at low collision energies.

There are five electronic states that correlate to the reagents C(^1^*D*)+D_2_, but the lowest two (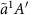 and 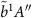) are the most relevant. As for the comparison with the excitation function measured in experiment, the inclusion of Renner–Teller effects is not expected to improve the agreement, since similar agreement is obtained (as shown in Fig. 2a,b) when the contribution of the 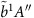 state is excluded. The three higher excited states can only participate in the reaction via the non-adiabatic pathways through the CIs, and their contributions are considered to be small in the collision energy range of this work. In particular, the minimum energy crossing point on the CI seam between the 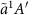 and 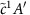 states is located at 12.4 kcal mol^−1^ as shown in Fig. 1a, below which the 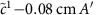 and 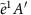 states are not accessible. Furthermore, although the rotational temperature of the experimental D_2_ beam^24^ is 100 K and only the D_2_(*j*=0) is considered here, our QCT results show that the difference between the ICS(*j*=0) and ICS(*j*=1, 2) is very small, thus the ortho-para nuclear spin effects could be neglected. Consequently, we think that, without further high-precision experimental work, it is unlikely to clarify the remaining small differences between the QD calculations on our PESs and experiment at low energies in Fig. 2.

Clearly the larger ICSs on the ZMB-a PES at low collision energies (Fig. 2b) will lead to larger rate coefficients. Supplementary Fig. 4 shows the initial state-specified rate coefficients as a function of temperature computed by us with the QD method on our 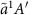 (ZMB-a) and 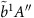 PESs. We see that, the 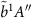 state makes a contribution of ∼38% to the overall reactivity, and our calculations suggest a larger rate coefficient than the experimental value of Sato *et al*.^23^ at room temperature. Our result is indirectly supported by a most recent experiment of Hickson *et al*^34^. In this experiment, they^34^ obtained a room temperature rate coefficient for the C(^1^*D*)+H_2_ reaction, which is larger than the upper limit reported by Sato *et al*.^23^ even when the experimental uncertainty is considered. Further experimental work is required to clarify the divergence^23^,^34^ in the measurements of rate coefficient.

### Product angular and state distributions

**Figure 4:**
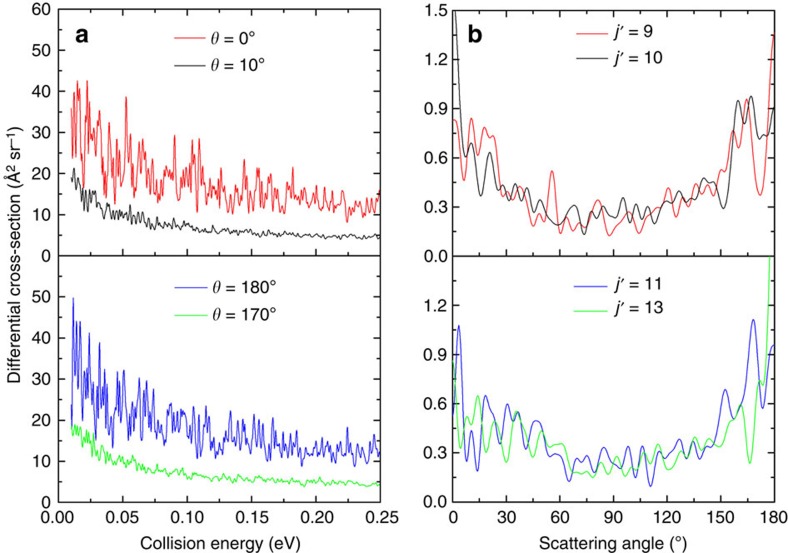
Quantum resonance and diffraction oscillations. These phenomena are predicted for the C(^1^*D*)+D_2_(*ν*=0, *j*=0)→CD(*ν*′, *j*′)+D reaction and the 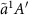 and 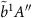 PESs constructed by us are used. (**a**) Energy dependence of differential cross-section with product states summed. (**b**) Product rotational state-resolved angular distributions at *ν*′=0 and the collision energy of 0.05 eV.

We also perform large-scale QD calculations on our 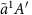 and 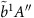 PESs with vdW saddles to predict more detailed scattering properties encoded in differential cross-sections (DCSs). In particular, dense resonance structures and quantum diffraction oscillations are revealed, which manifest themselves as two kinds of different oscillation patterns in calculated DCSs (Fig. 4). We present the energy dependence of DCS in Fig. 4a, and we see that the DCSs near the two extreme angles (0° and 180°) display similar highly oscillatory structures, indicating densely distributed sharp resonances, which result from the numerous long-lived quasibound states supported by the deep well. We can also notice that the amplitude of the oscillations is reduced quickly when the scattering angle deviates from 0° or 180°. Meanwhile, we show the angular dependence of the product rotational state-resolved DCS at a representative collision energy in Fig. 4b, which exhibits an interesting oscillation pattern with peaks. This phenomenon may be caused by quantum interferences between partial waves, which appears to be similar to the diffraction oscillations just observed experimentally in the H+D_2_ reaction by Zare and coworkers using the photoloc technique^40^,^41^. However, they are of different nature, and as demonstrated by Zare and coworkers^41^, the latter is caused by well separated sets of total angular momentum, leading to scattering at the same angles.

**Figure 5:**
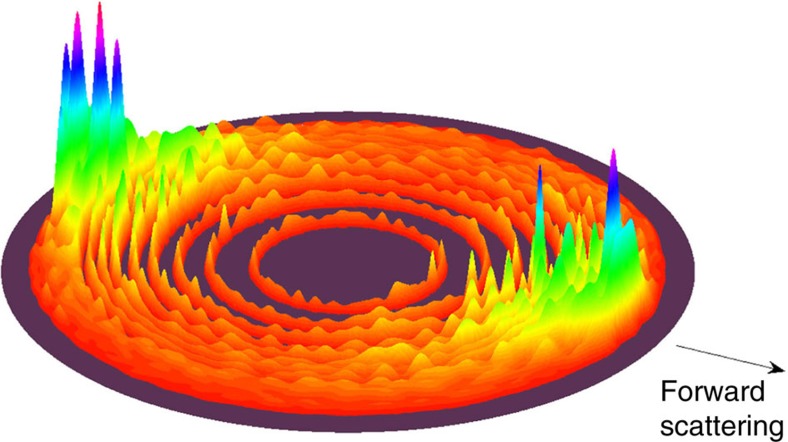
Three-dimensional CD product flux in the centre-of-mass velocity space. This is calculated on our 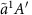 and 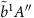 PESs and at the collision energy of 0.038 eV for the C(^1^*D*)+D_2_(*ν*=0, *j*=0) reaction. The forward scattering (*θ*=0°) is defined along the C atom reactant beam direction.

In addition, we present a three-dimensional CD product flux surface plot at the collision energy of 0.038 eV in Fig. 5, in which the concentric circles correspond to different ro-vibrational states (*ν*′, *j*′) of the CD product. By integrating over the angular distribution shown in Fig. 5, we can obtain the rotational state distribution of the CD product, which is found to be in very good agreement with the available experimental measurements^23^ (Supplementary Fig. 5). We can also see that the product angular distribution is dominated by scattering in both the forward and backward directions with roughly forward–backward symmetry; this trend persists over the whole energy range, according with the complex-forming mechanism. In particular, the calculated results on our 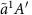 and 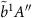 PESs at higher collision energies are consistent with the observations of the crossed-beam experiments^24^,^25^, in which the product angular distributions are found to be forward–backward symmetric. It should be noted that the calculated DCSs on the 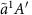 PES, either RKHS or ZMB-a, are nearly forward–backward symmetric^25^,^27^,^36^; however previous calculations show that the addition of the contribution of the 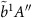 BJHL PES^37^ leads to a forward bias in DCS^38^,^42^, in contradiction to experiment, indicating that the present 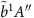 PES constructed by us is more accurate than the BJHL PES.

### Product vibrational branching ratio

**Figure 6:**
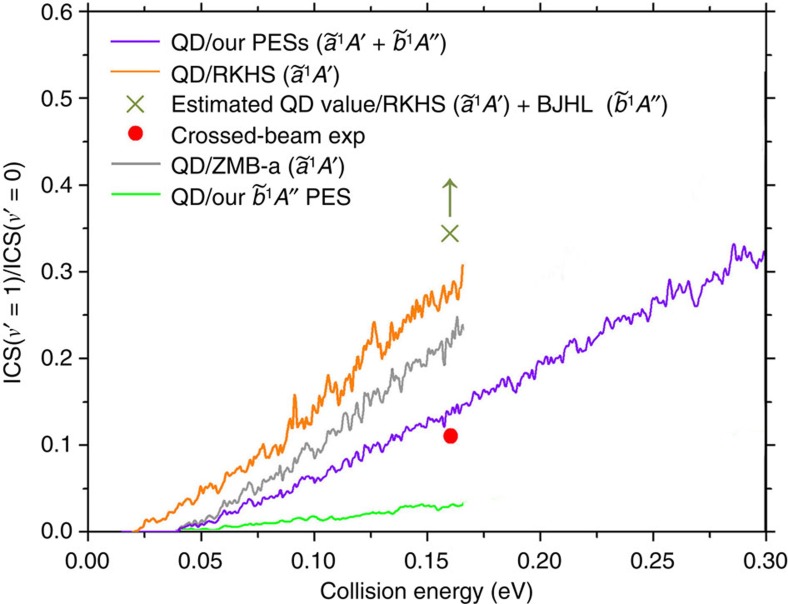
Product vibrational branching ratio as a function of collision energy. This is defined as the ratio between the integral cross-section (ICS(*ν*′=1)) and ICS(*ν*′=0) for the C(^1^*D*)+D_2_(*ν*=0, *j*=0)→CD(*ν*′)+D reaction. Accurate quantum dynamics (QD) results calculated on our 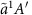 and 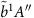 PESs are compared with the available crossed-beam experimental value^25^, along with our QD results calculated on the RKHS PES and the roughly estimated lower limit of the QD value on the RKHS (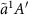) and BJHL (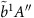) PESs. The corresponding contributions of our 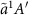 and 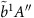 PESs are also shown, respectively.

Further evidence about the advantage of our PESs and the importance of the excited 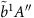 PES is presented in Fig. 6, which is about the product CD vibrational branching ratio defined as ICS(*ν*′=1)/ICS(*ν*′=0). We see that the present QD result calculated on our 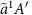 and 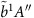 PESs is in very good agreement with the available experimental value obtained by Balucani *et al*.^25^ using the crossed-beam technique; meanwhile, the addition of the contribution of our excited 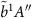 PES evidently reduces the total vibrational branching ratio and improves the agreement with experiment. In contrast, the present QD result calculated on the RKHS PES shows a much larger value, and the addition of the contribution of the BJHL (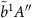) PES would evidently further increase the vibrational branching ratio according to the previous calculations^38^,^42^. A schematic lower limit of the QD branching ratio including contributions from both the RKHS (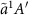) and BJHL (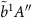) PESs is also indicated in Fig. 6, and the contribution from the BJHL PES is indirectly estimated from the previous QD calculations for the C(^1^*D*)+H_2_ reaction^38^. Actually, the previous calculations on the BJHL excited PES yielded significantly larger vibrational branching ratio^38^,^42^ than that on the singlet-ground-state PES (BHL or RKHS), and it was concluded by Casavecchia and coworkers^38^ that the participation of the BJHL surface leads to an excessive contribution from the product CH(*ν*′=1) channel, well above the possible experimental uncertainty. As shown in Supplementary Fig. 6, the topological feature of our 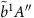 PES is very different from that of the BJHL PES (Supplementary Discussion), and this should be the main cause of the large divergence. Our classical trajectory analysis further indicates that the nonstatistical and complex reaction mechanisms related to the shallow well structure on our 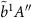 PES are responsible for the highly cold CD vibrational distribution. It should be mentioned that the D_2_ beam used in the experiment^25^ has a relative rotational population, and slightly larger theoretical values may be obtained than those shown in Fig. 6 when the initial D_2_ rotational excitation is considered. We have calculated the vibrational branching ratio using the QCT method by averaging the contributions from different rotational states according to the experimental condition, and the obtained value is somewhat larger, but still in very good agreement with experiment. We think that the present theoretical values on our 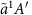 and 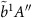 PESs are within the experimental error limit according to the relevant statements of Casavecchia and coworkers^25^,^38^.

## Discussion

Accurate QD and QCT calculations studying vdW interactions and excited-state PES contributions in the C(^1^*D*)+D_2_ reaction have been reported, using the 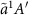 and 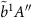 *ab initio* PESs constructed by us, the latter of which is reported in this work. Our PESs have entrance and exit-channel vdW saddles, and are unique in the accurate description of the vdW regions. The QD calculations using our 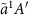
and 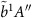 PESs yield dynamical quantities in very good agreement with experiment, including the excitation function, product vibrational branching ratio and rotational state distribution; the obtained product angular distributions are consistent with those found in the crossed-beam experiments, which are forward–backward symmetric. These were not achieved on the 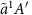 and 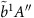 PESs reported by others. Furthermore, interesting diffraction oscillations and numerous sharp resonances are predicted.

The importance of both vdW saddle and excited 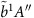 PES in reaction dynamics is underscored. Our calculations indicate that the 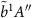 excited-state PES plays a significant role in the C(^1^*D*)+D_2_ reaction dynamics. The present QD calculations on our ZMB-a PES yield reasonable cross-sections supported by the recent crossed-beam experiment, whereas the same calculations on the earlier RKHS PES with the vdW well produce much smaller cross-sections at low collision energies, at which detailed analyses with the QCT method reveal that the anisotropic interactions around the vdW saddle point on ZMB-a lead to a D–D torsion and then C sideways insertion reaction mechanism, whereas the vdW well on RKHS inhibits the reactivity.

The concept of ‘vdW saddle' proposed in this work may have general importance, and our conjecture is, the vdW saddle originates from the nature of the polyatomic complex-forming reactions that involve deep wells. The shallow entrance-channel vdW well has played an important role in acquiring a deeper understanding of the direct reactions that are dominated by activation barriers; it is our hope that the vdW saddle will prove to be a useful concept in the study of complex-forming reactions that are dominated by deep wells.

## Methods

### *Ab initio* calculations and PES

All *ab initio* calculations were performed with the MOLPRO package^43^. The internally contracted multireference configuration interaction^44^, and five-state-averaged complete active space self-consistent field methods^45^ were used. Five reference states were used for generating the internally contracted pairs. The active space consists of six electrons distributed among seven orbitals. The basis set used is the Dunning's correlation consistent quadruple-zeta basis augmented by diffuse functions (aug-cc-pVQZ)^46^. A highly accurate global PES for the 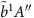 state of the title system was constructed based upon 6,639 symmetry unique energy points obtained from our *ab initio* calculations. Accurate analytical fits were generated using many-body expansions with the permutationally invariant polynomials, and the scheme of PES construction is similar to that described in our recent work^35^, where a highly accurate global ground-state (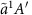) PES called ZMB-a was constructed. The root-mean-square error of the global fit is 0.42 kcal mol^−1^ for energy points below 15 kcal mol^−1^ relative to the C(^1^*D*)+D_2_ asymptote, though the fitting errors in dynamically important regions are much lower. To illustrate the global feature of our 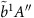 surface, several typical contour plots as functions of Jacobi coordinates *R* and *r* at different chosen angles are shown in Supplementary Fig. 6. Further details about our 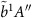 surface and discussion are given in Supplementary Methods and Supplementary Discussion.

### QD and QCT calculations

The QD calculations including all Coriolis couplings were performed using the real-wave packet approach as implemented in the DIFFREALWAVE code^47^. Numerical details are given in Supplementary Table 3, and numerous test calculations were performed to check the convergence. All partial wave contributions up to *J*=50 were included, yielding converged ICSs and DCSs for collision energies up to ∼0.3 eV. About 120,000 iteration steps were implemented for each partial wave calculation, which were reduced at high *J* values. The QCT calculations were performed using a modified version of the VENUS96 code^48^,^49^ customized to incorporate the PESs. Batches of 15,000–30,000 trajectories were calculated for each set of initial conditions with an integration time step of 0.02 fs, which guarantees an energy conservation of better than 1 in 10^6^. The trajectories were initiated at the C(^1^*D*)+D_2_ asymptote with an atom–diatom separation of 15.0 bohr. To fix the zero-point energy problem, the Gaussian binning^50^ approach was used with a full width at half maximum of 0.1 in the Gaussian weighting function. Further details about the methods and calculations are given in Supplementary Methods and Supplementary References.

### Data availability

The data that support the findings of this study are available within the article (and its Supplementary Information files) and from the corresponding author on request.

## Additional information

**How to cite this article:** Shen, Z. *et al*. Dynamical importance of van der Waals saddle and excited potential surface in C(^1^*D*)+D_2_ complex-forming reaction. *Nat. Commun.* **8,** 14094 doi: 10.1038/ncomms14094 (2017).

**Publisher's note**: Springer Nature remains neutral with regard to jurisdictional claims in published maps and institutional affiliations.

## Supplementary Material

Supplementary Information Supplementary Figures, Supplementary Tables, Supplementary Discussion, Supplementary Methods and Supplementary References.

## References

[b1] Levine R. D. *Molecular Reaction Dynamics* (Cambridge Univ. Press, 2005).

[b2] Skouteris D. . van der Waals interactions in the Cl+HD reaction. Science 286, 1713–1716 (1999). 10576733 10.1126/science.286.5445.1713

[b3] Tezcan F. A., Crane B. R., Winkler J. R. & Gray H. B. Electron tunneling in protein crystals. Proc. Natl Acad. Sci. USA 98, 5002–5006 (2001). 11296248 10.1073/pnas.081072898 PMC33153

[b4] Zhang W., Kawamata H. & Liu K. CH stretching excitation in the early barrier F+CHD_3_ reaction inhibits CH bond cleavage. Science 325, 303–306 (2009). 19608914 10.1126/science.1175018

[b5] Czakó G. & Bowman J. M. CH stretching excitation steers the F atom to the CD bond in the F+CHD_3_ reaction. J. Am. Chem. Soc. 131, 17534–17535 (2009). 19908862 10.1021/ja906886z

[b6] Wang F., Lin J.-S. & Liu K. Steric control of the reaction of CH stretch-excited CHD_3_ with chlorine atom. Science 331, 900–903 (2011). 21330543 10.1126/science.1199771

[b7] Czakó G. & Bowman J. M. Dynamics of the reaction of methane with chlorine atom on an accurate potential energy surface. Science 334, 343–346 (2011). 22021853 10.1126/science.1208514

[b8] Xie T., Wang D., Bowman J. M. & Manolopoulos D. E. Resonances in the O(^3^P)+HCl reaction due to van der Waals minima. J. Chem. Phys. 116, 7461–7467 (2002).

[b9] Bian W. & Werner H.-J. Global *ab initio* potential energy surfaces for the ClH_2_ reactive system. J. Chem. Phys. 112, 220–229 (2000).

[b10] Cao J. . Quasiclassical trajectory study of H+SiH_4_ reactions in full-dimensionality reveals atomic-level mechanisms. Proc. Natl Acad. Sci. USA 106, 13180–13185 (2009). 19666504 10.1073/pnas.0903934106 PMC2726355

[b11] Li J., Jiang B. & Guo H. Enhancement of bimolecular reactivity by a pre-reaction van der Waals complex: the case of F+H_2_O→HF+HO. Chem. Sci. 4, 629–632 (2013).

[b12] Hase W. L. Simulations of gas-phase chemical reactions: applications to S_N_2 nucleophilic substitution. Science 266, 998–1002 (1994). 17779941 10.1126/science.266.5187.998

[b13] Orr-Ewing A. J. & Zare R. N. Orientation and alignment of reaction products. Annu. Rev. Phys. Chem 45, 315–366 (1994).

[b14] Simpson W. R., Rakitzis T. P., Kandel S. A., Orr-Ewing A. J. & Zare R. N. Reaction of Cl with vibrationally excited CH_4_ and CHD_3_: state-to-state differential cross sections and steric effects for the HCl product. J. Chem. Phys. 103, 7313–7335 (1995).

[b15] Clary D. C. Fast chemical reactions: theory challenges experiment. Annu. Rev. Phys. Chem. 41, 61–90 (1990).

[b16] Levine R. D. The chemical shape of molecules: an introduction to dynamical stereochemistry. J. Phys. Chem. 94, 8872–8880 (1990).

[b17] Ospelkaus S. . Quantum-state controlled chemical reactions of ultracold potassium-rubidium molecules. Science 327, 853–857 (2010). 20150499 10.1126/science.1184121

[b18] Kim J. B. . Spectroscopic observation of resonances in the F+H_2_ reaction. Science 349, 510–513 (2015). 26228142 10.1126/science.aac6939

[b19] Balucani N., Capozza G., Leonori F., Segoloni E. & Casavecchia P. Crossed molecular beam reactive scattering: From simple triatomic to multichannel polyatomic reactions. Int. Rev. Phys. Chem. 25, 109–163 (2006).

[b20] Aoiz F. J., Bañares L. & Herrero V. J. Dynamics of insertion reactions of H_2_ molecules with excited atoms. J. Phys. Chem. A 110, 12546–12565 (2006). 17107104 10.1021/jp063815o

[b21] González-Lezana T. Statistical quantum studies on insertion atom-diatom reactions. Int. Rev. Phys. Chem. 26, 29–91 (2007).

[b22] Guo H. Quantum dynamics of complex-forming bimolecular reactions. Int. Rev. Phys. Chem. 31, 1–68 (2012).

[b23] Sato K., Ishida N., Kurakata T., Iwasaki A. & Tsunashima S. Reactions of C(^1^D) with H, HD and D_2_: kinetic isotope effect and the CD/CH branching ratio. Chem. Phys. 237, 195–204 (1998).

[b24] Liu K. Excitation functions of elementary chemical reactions: a direct link from crossed-beam dynamics to thermal kinetics? Int. Rev. Phys. Chem. 20, 189–217 (2001).

[b25] Balucani N. . Dynamics of the C(^1^D)+D_2_ reaction: a comparison of crossed molecular-beam experiments with quasiclassical trajectory and accurate statistical calculations. J. Chem. Phys. 122, 234309 (2005). 16008443 10.1063/1.1930831

[b26] Bussery-Honvault B., Honvault P. & Launay J.-M. A study of the C(^1^D)+H_2_→CH+H reaction: global potential energy surface and quantum dynamics. J. Chem. Phys. 115, 10701–10708 (2001).

[b27] Bañares L., Aoiz F. J., Vázquez S. A., Ho T.-S. & Rabitz H. Quasi-classical trajectory calculations on a fast analytic potential energy surface for the C(^1^D)+H_2_ reaction. Chem. Phys. Lett. 374, 243–251 (2003).

[b28] Joseph S. & Varandas A. J. C. Accurate double many-body expansion potential energy surface for the lowest singlet state of methylene. J. Phys. Chem. A 113, 4175–4183 (2009). 19296630 10.1021/jp810600r

[b29] Joseph S., Caridade P. J. S. B. & Varandas A. J. C. Quasiclassical trajectory study of the C(^1^D)+H_2_ reaction and isotopomeric variants: kinetic isotope effect and CD/CH branching ratio. J. Phys. Chem. A 115, 7882–7890 (2011). 21644798 10.1021/jp2032912

[b30] Lin S. & Guo H. Quantum wave packet studies of the C(^1^D)+H_2_→CH+H reaction: integral cross section and rate constant. J. Phys. Chem. A 108, 2141–2148 (2004).

[b31] Rackham E. J., Gonzalez-Lezana T. & Manolopoulos D. E. A rigorous test of the statistical model for atom-diatom insertion reactions. J. Chem. Phys. 119, 12895–12907 (2003). 10.1063/1.2723067 17477580

[b32] Aoiz F. J., González-Lezana T. & Rábanos V. S. A comparison of quantum and quasiclassical statistical models for reactions of electronically excited atoms with molecular hydrogen. J. Chem. Phys. 129, 094305 (2008). 19044868 10.1063/1.2969812

[b33] Defazio P., Bussery-Honvault B., Honvault P. & Petrongolo C. Nonadiabatic quantum dynamics of C(^1^D)+H_2_→CH+H: coupled-channel calculations including Renner-Teller and Coriolis terms. J. Chem. Phys. 135, 114308 (2011). 21950863 10.1063/1.3636083

[b34] Hickson K. M., Loison J.-C., Guo H. & Suleimanov Y. V. Ring-polymer molecular dynamics for the prediction of low-temperature rates: an investigation of the C(^1^D)+H_2_ reaction. J. Phys. Chem. Lett. 6, 4194–4199 (2015). 26538033 10.1021/acs.jpclett.5b02060

[b35] Zhang C., Fu M., Shen Z., Ma H. & Bian W. Global analytical *ab initio* ground-state potential energy surface for the C(^1^D)+H_2_ reactive system. J. Chem. Phys. 140, 234301 (2014). 24952535 10.1063/1.4881896

[b36] Shen Z., Cao J. & Bian W. Quantum mechanical differential and integral cross sections for the C(^1^D)+H_2_(*ν*=0, *j*=0)→CH(*ν*′, *j*′)+H reaction. J. Chem. Phys. 142, 164309 (2015). 25933766 10.1063/1.4919406

[b37] Bussery-Honvault B., Julien J., Honvault P. & Launay J.-M. Global 1^1^*A*″ potential energy surface of CH_2_ and quantum dynamics of a sideways insertion mechanism for the C(^1^D)+H_2_→CH(^2^II)+H reaction. Phys. Chem. Chem. Phys. 7, 1476–1481 (2005). 19787971 10.1039/b419000a

[b38] Balucani N. . Dynamics of the C(^1^D)+H_2_ reaction: A comparison of crossed molecular beam experiments with quantum mechanical and quasiclassical trajectory calculations on the first two singlet (1^1^A′ and 1^1^A″) potential energy surfaces. Mol. Phys. 108, 373–380 (2010).

[b39] Aoiz F. J., Sáez Rábanos V., González-Lezana T. & Manolopoulos D. E. A statistical quasiclassical trajectory model for atom-diatom insertion reactions. J. Chem. Phys. 126, 161101 (2007). 17477580 10.1063/1.2723067

[b40] Jambrina P. G. . Quantum interference between H+D_2_ quasiclassical reaction mechanisms. Nat. Chem. 7, 661–667 (2015). 26201743 10.1038/nchem.2295

[b41] Jambrina P. G., Aldegunde J., Aoiz F. J., Sneha M. & Zare R. N. Effects of reagent rotation on interferences in the product angular distributions of chemical reactions. Chem. Sci. 7, 642–649 (2016). 10.1039/c5sc03373j PMC5523120 28791109

[b42] Honvault P., Bussery-Honvault B., Launay J.-M., Aoiz F. J. & Bañares L. Quantum mechanical and quasiclassical trajectory scattering calculations for the C(^1^D)+H_2_ reaction on the second excited 1^1^*A*″ potential energy surface. J. Chem. Phys. 124, 154314 (2006). 16674233 10.1063/1.2187007

[b43] Werner H.-J. . MOLPRO, Version 2006.1, a package of *ab initio* programs. Available at http://www.molpro.net.

[b44] Werner H.-J. & Knowles P. J. An efficient internally contracted multiconfiguration-reference configuration interaction method. J. Chem. Phys. 89, 5803–5814 (1988).

[b45] Werner H.-J. & Knowles P. J. A second order multiconfiguration SCF procedure with optimum convergence. J. Chem. Phys. 82, 5053–5063 (1985).

[b46] Dunning T. H. Gaussian basis sets for use in correlated molecular calculations. I. The atoms boron through neon and hydrogen. J. Chem. Phys. 90, 1007–1023 (1989).

[b47] Hankel M., Smith S. C., Gray S. K. & Balint-Kurti G. G. DIFFREALWAVE: A parallel real wavepacket code for the quantum mechanical calculation of reactive state-to-state differential cross sections in atom plus diatom collisions. Comput. Phys. Commun. 179, 569–578 (2008).

[b48] Hase W. L. . Venus96: a general chemical dynamics computer program. QCPE Bull. 16, 671 (1996).

[b49] Peslherbe G. H., Wang H. & Hase W. L. Monte Carlo sampling for classical trajectory simulations. Adv. Chem. Phys. 105, 171–201 (1999).

[b50] Bonnet L. & Rayez J. Quasiclassical trajectory method for molecular scattering processes: necessity of a weighted binning approach. Chem. Phys. Lett. 277, 183–190 (1997).

