# Supplementary material for: Dynamical importance of van der Waals saddle and excited potential surface in C(1D)+D2 complex-forming reaction
Source: Nat Commun. 2017 Jan 17;8:14094. doi: 10.1038/ncomms14094 (PMC5247604; doi:10.1038/ncomms14094)
Supplement: Supplementary Information — Supplementary Figures, Supplementary Tables, Supplementary Discussion, Supplementary Methods and Supplementary References. [file ncomms14094-s1.pdf]

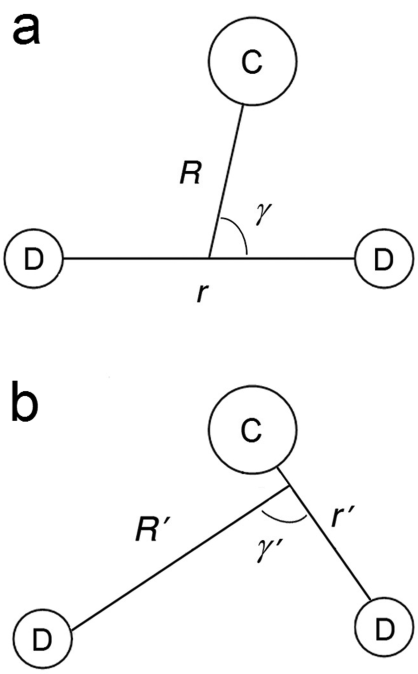

Supplementary Figure 1. **The coordinate definitions**. (a) Reactant Jacobi coordinates; (b) product Jacobi coordinates.

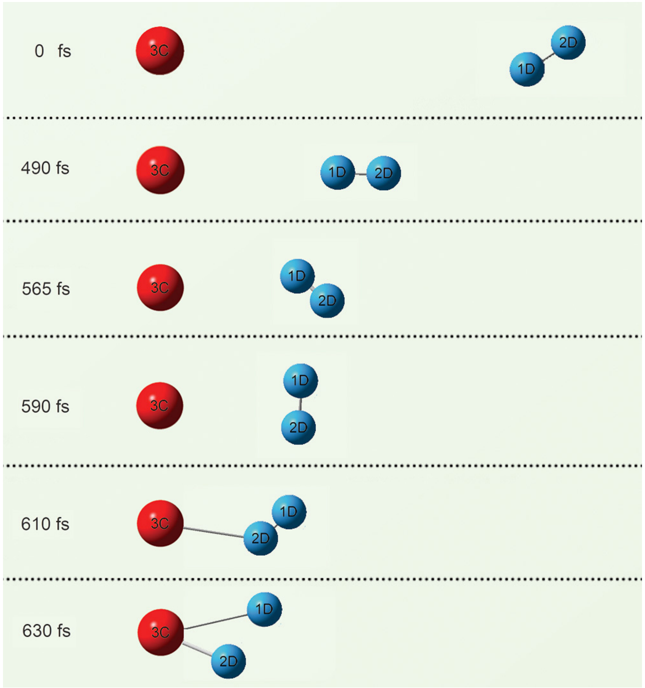

Supplementary Figure 2. **Snapshots of a typical reactive trajectory**. The trajectory is in the process of first 630 fs of the $C(^1D)+D_2(\nu = 0, j = 0)$ approaching at the collision energy of 0.005 eV.

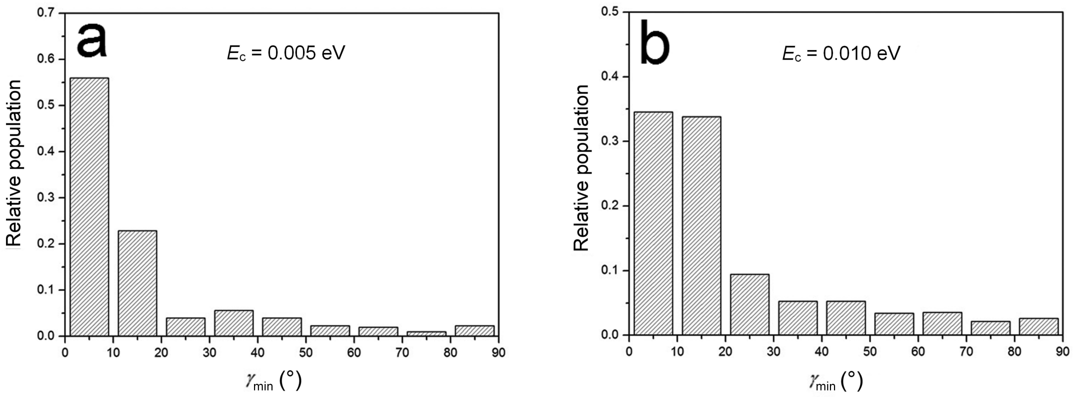

Supplementary Figure 3. **Relative population of reactive trajectories in different $\gamma_{\mathbf{min}}$ bins**. All the trajectories are calculated at the collision energies (E_c) of (a) 0.005 eV and (b) 0.010 eV, with the initiated γ ranging from 0 to 90.0° and the impact parameter b being sampled from $b = b_{\text{max}}\beta^{1/2}$, where β is a random number in the $[0, 1]$ interval and b_{max} is the maximum impact parameter, and for a given trajectory γ is defined as the Jacobi angle of the trajectory configuration (Supplementary Fig. 1a) at a certain time. The γ_{min} is the minimum value of γ when the trajectory is in the entrance valley.

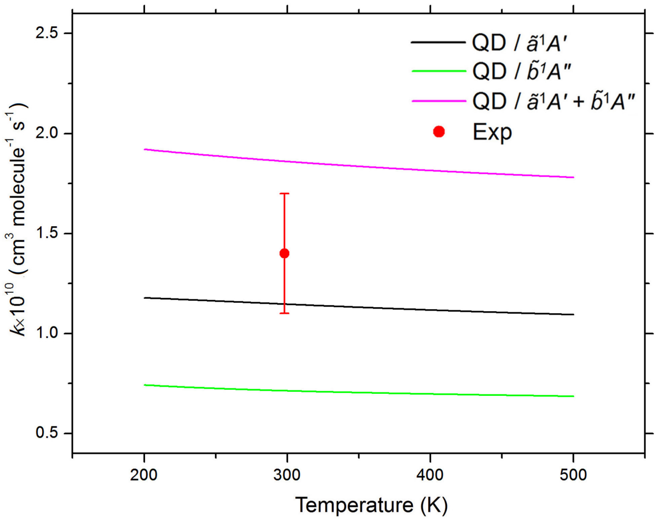

Supplementary Figure 4. Temperature dependence of rate coefficient k for the $C(^1D)$+D_2(ν=0,j=0) reaction. Accurate quantum dynamics (QD) results calculated on our $\tilde{a}^1A'$ and $\tilde{b}^1A''$ potential energy surfaces (PESs) (in pink line) are presented, along with the experimental value (in red circle) [1] at room temperature. The corresponding contributions of the $\tilde{a}^1A'$ (in black line) and $\tilde{b}^1A''$ (in green line) PESs are also shown, respectively.

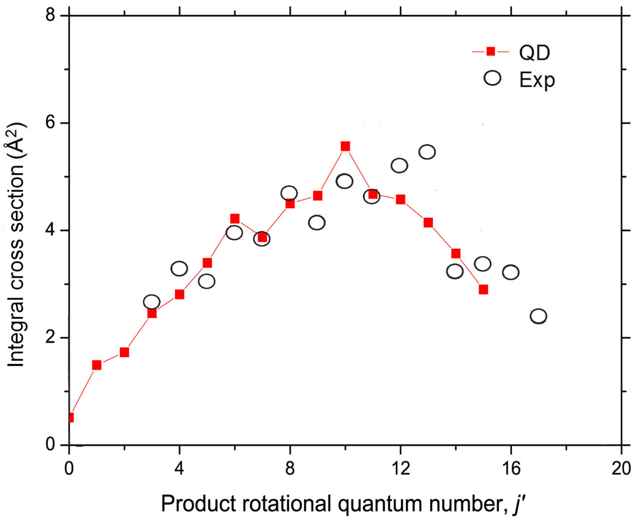

Supplementary Figure 5. **Product rotational state distributions for the C(^1D)+D$_2$($\nu = 0, j = 0$)→CD($\nu' = 0, j'$)+D reaction**. The $\tilde{a}^1A'$ and $\tilde{b}^1A''$ PESs constructed by us are used. The present quantum dynamical (QD) results calculated at the collision energy of 0.038 eV are compared with the experimental measurements [1], which are normalized with the QD result at $j' = 7$.

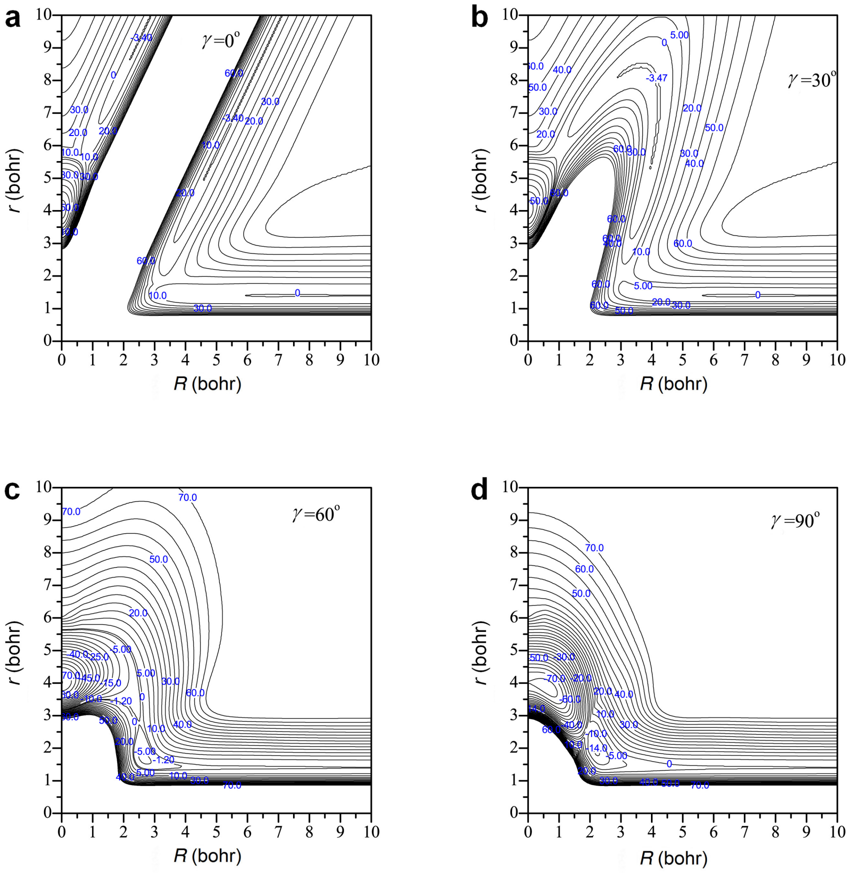

Supplementary Figure 6. **Contour plots of our $\tilde{b}^1A''$ PES**. The reactant Jacobi coordinates (Supplementary Fig. 1a) are used, with γ fixed at (a) 0.0°, (b) 30.0°, (c) 60.0° and (d) 90.0°. The contours are in kcal mol^{-1} relative to the reagent asymptote.

Supplementary Table 1. Properties of entrance-channel van der Waals saddle points. The stationary points in the C(^1D)+D_2 reactive system are optimized using the five-state *ic*MRCI+Q/aug-cc-pVQZ method (bond length in bohr, bond angle in degree, frequency in cm^{-1}).[a]

State	Geometries	Frequencies[b]
$\tilde{a}^1A'$	$R_{CD} = 5.98$	$\omega_1 = 3102.61$
	$R_{DD} = 1.404$	$\omega_2 = 67.92$
	$\angle CDD = 180.0$	$\omega_3 = 631.90i$
		$\omega_4 = 631.78i$
$\tilde{b}^1A''$	$R_{CD} = 6.06$	$\omega_1 = 3106.09$
	$R_{DD} = 1.403$	$\omega_2 = 57.58$
	$\angle CDD = 180.0$	$\omega_3 = 345.17i$
		$\omega_4 = 345.13i$

[a] The optimization calculations are performed using internally contracted multireference configuration interaction (*ic*MRCI) method [2, 3], in which orbitals are obtained from the five-state-averaged complete active space self-consistent field calculation [4, 5] and five reference states are used for generating the internally contracted pairs. The Davidson correction ($+Q$) is employed to include the correlation energy due to higher excitations. The active space consists of 6 electrons distributed among 7 orbitals. The basis set used is aug-cc-pVQZ [6, 7]. All *ab initio* calculations are performed with the MOLPRO suite of *ab initio* programs [8].

[b] Frequencies multiplied by i are imaginary frequencies.

Supplementary Table 2. **Properties of stationary points on our $\tilde{b}^1A''$ potential energy surface**. For comparison those from *ab initio* calculations, on the BJHL potential energy surface (PES) and from experiment are also shown (bond length in bohr, bond angle in degree, and energy [a] in kcal mol^{-1}).

Property		Our work		BJHL[b]	Expt.
		ab initio	$\tilde{b}^1A''$PES		
$CD_2(\tilde{b}^1A'')$	R_{CD}	2.032	2.032	2.03	2.052 [c]
	∠DCD	143.12	143.44	141.0	139.4±19.0[c]
	E	-77.71	-77.71	-79.9	
$C(^1D)+D_2$	R_{DD}	1.402	1.402	1.40	1.401[d]
$CD(^2\Pi)+D$	R_{CD}	2.116	2.117	2.12	2.116[d]
	E	-3.473	-3.473	-3.86	-3.69±0.25[e]
Shallow minimum of the highly bent C_{2v} geometry	R	2.20	2.215		
	r	1.90	1.886		
	γ	90.0	90.0		
	E	-14.372	-14.359		
van der Waals saddle point in the entrance channel	R_{CD}	6.052	6.044		
	R_{DD}	1.403	1.404		
	∠CDD	180.0	180.0		
	E	-0.221	-0.225		
van der Waals saddle point in the CD-D exit channel	R_{CD}	2.116	2.117		
	R_{DD}	5.904	5.908		
	∠CDD	180.0	180.0		
	E[f]	-0.074	-0.069		
van der Waals saddle point in the DC-D exit channel	R_{CD1}	2.118	2.116		
	R_{CD2}	7.562	7.556		
	∠DCD	180.0	180.0		
	E[f]	-0.059	-0.056		

[a] The energy of the $C(^1D)+D_2$ asymptote is taken to be zero.
[b] The BJHL PES of Ref. [9].
[c] Experimental result from Ref. [10].
[d] Experimental result from Ref. [11].
[e] Deduced from Refs. [11] and [12].
[f] Relative to the energy of the CD+D asymptote.

Supplementary Table 3. **Numerical parameters used in the wave packet calculation.**[a]

Scattering coordinate (R') range	0−21.0
Number of grid points in R'	224
Internal coordinate (r') range	0.5−19.5
Number of grid points in r'	215
Number of angular grid points	160
Absorption region length in R' (r')	5.0 (5.0)
Absorption strength in R' (r')	0.1 (0.1)
Center of initial wave packet (R_0)	12.0
Width of the wave packet, α	8.0
Smoothing of the wave packet, β	0.5
Initial translational energy	0.15 eV
Analysis line, R'_∞	16.0
Cutoff energy, V_{cut}	0.5

[a] All quantities are given in atomic units unless stated otherwise. The product Jacobi coordinates R', r' and γ' (see Supplementary Fig. 1b) are used.

III. SUPPLEMENTARY DISCUSSION

A. Potential energy surface

There are three van der Waals (vdW) saddles on the $\tilde{b}^1A''$ potential energy surface (PES) constructed by us, and the saddle points are located at a linear C-DD geometry in the entrance channel, and at linear CD-D and DC-D geometries in the exit channel, respectively. We calculate more than 100 *ab initio* points around each vdW extremal point, and then fit these *ab initio* points with great care; our fitting basically reproduces our *ab initio* calculational values of the location and energy for each vdW saddle point. For example, in the entrance channel, our surface has a C-DD vdW saddle point at R_{CD} = 6.044 bohr and R_{DD} = 1.404 bohr with an energy of -0.225 kcal mol^{-1} relative to the C(^1D)+D_2 asymptote, which are quite close to our *ab initio* calculational values (R_{CD} = 6.052 bohr, R_{DD} = 1.403 bohr, and E = -0.221 kcal mol^{-1}). Please note that these *ab initio* calculational values are obtained directly from the calculated densely distributed *ab initio* points, and are slightly different from those obtained with optimization procedures, which are reported in Supplementary Table 1 along with imaginary frequencies at the saddle point. Furthermore, in the exit channel, our surface possesses a vdW saddle with the linear CD-D complex being 0.069 kcal mol^{-1} lower than the CD+D asymptote and at R_{CD} = 2.117 bohr, R_{DD} = 5.908 bohr, and has another DC-D vdW saddle point at R_{CD1} = 2.116 bohr, R_{CD2} = 7.556 bohr with an energy of -0.056 kcal mol^{-1} relative to the CD+D asymptote.

Furthermore, we see from Supplementary Table 2 that, the reaction enthalpy $\Delta_{\mathrm{r}}H_{\mathrm{e}}$ from our $\tilde{b}^1A''$ PES is in very good agreement with the value of our *ab initio* calculations, which is within the experimental error limit, but may be slightly underestimated due to the neglect of core-correlation effects of the 1s electrons in the C atom, whereas that from the BJHL surface (-3.86 kcal mol^{-1}) may be somewhat overestimated. The global $\tilde{b}^1A''$ surface exhibits a deep well (-77.71 kcal mol^{-1} relative to the C(^1D)+D_2 asymptote) but is shallower than that of the ZMB-a surface (-100.54 kcal mol^{-1}), which indicates that the $\tilde{b}^1A''$ surface may allow more nonstatistical behaviors than the ZMB-a surface. The energy of the global minimum at

$R_{\rm CD}$ = 2.032 bohr and ∠DCD = 143.44° is higher by about 2.19 kcal mol^{-1} than that of the BJHL surface although the geometry parameters from the two PESs are quite similar.

Contour plots in various PES regions are checked carefully, and we think that our $\tilde{b}^1A''$ surface can describe all dynamically important regions properly. Typical contour plots of our surface as a function of reactant Jacobi coordinates (see Supplementary Fig. 1a) R and r with $\gamma = 0°, 30°, 60°$ and 90° are shown in Supplementary Fig. 6. We see from Supplementary Fig. 6a that, for the collinear geometries ($\gamma = 0°$), our surface presents high barriers in the reactant (12.43 kcal mol^{-1}) and product (9.26 kcal mol^{-1}) arrangements. The corresponding barriers can also be found on the BJHL surface [9], but the barrier heights for BJHL, which are 9.9 and 4.43 kcal mol^{-1} for the reactant and product arrangements respectively, are much lower than those for our surface. Supplementary Figure 6b shows that, when γ increases from 0° to 30°, the two barrier heights become lower, which are around 7.0 and 2.0 kcal mol^{-1} in the reactant and product arrangements, respectively.

More importantly, we can see that a shallow well appears in the entrance channel along the perpendicular insertion direction (see Supplementary Fig. 6d) and this shallow well is connected with the deep well by a small barrier. This kind of structure on our surface is very different from that displayed on the BJHL PES [9], which presents an extremely high barrier (83.0 kcal mol^{-1}) in the contour plot for $\gamma = 90°$. Obviously this significant difference can not be attributed to the fitting procedure but should result from the *ab initio* calculations for the construction of the two PESs. As described in the Methods section, our *ab initio* calculations are much larger than those for the construction of the BJHL PES, and we notice that the single-state multireference configuration interaction calculations [9] are performed for BJHL, which may encounter problems due to the mixing of different electronic states. In the present calculations five reference states are used for generating the internally contracted pairs in the *ic*MRCI procedure [2, 3], and we think this is important to get a realistic PES topological structure along the perpendicular insertion direction (Supplementary Fig. 6d). Clearly, these topological differences

between the BJHL and our surfaces may lead to different reaction mechanisms.

B. Relative population in Supplementary Fig. 3

We make detailed analyses for all the calculated reactive trajectories, which are initiated with different impact parameters b and Jacobi angles γ (0–90.0°). For most of the trajectories the γ is actually varying (we call it as D-D torsion) during the process of C(^1D)+D_2 approaching because of the reorientation effects induced by the vdW interactions of ZMB-a. For each trajectory we examine the change of the γ value in the vdW region and find the minimum (denoted as γ_{min}) to determine whether the D-D torsion occurs. Therefore, for a given trajectory a small γ_{min} value reflects that this trajectory has a nearly collinear configuration at a certain time (just like the trajectory in Supplementary Fig. 2 at the time of 490 fs), and we use the γ_{min} to distinguish between different mechanisms in the C(^1D)+D_2 reaction. Obviously, trajectories undergoing the D-D torsion then C sideways insertion reaction mechanism will have small γ_{min} values (roughly $\gamma_{\mathrm{min}}<10°$) owing to the linear C-DD vdW saddle-point structure in the entrance valley. As shown in Supplementary Fig. 3, most reactive trajectories are found to have small γ_{min} values, indicating that the D-D torsion then C sideways insertion reaction mechanism proposed in this work is the major mechanism at low collision energies.

IV. SUPPLEMENTARY METHODS

A. Potential energy surface construction

A global *ab initio* PES for the $\tilde{b}^1A''$ state of the C(^1D)+D_2 reactive system was constructed. The regions around conical intersections were fitted separately and then merged with the well and asymptote regions using switching functions to produce the global surface. The forms of those switching functions were carefully chosen to make sure that they always have continuous first derivatives and are permutationally invariant. The resulting global surface is very smooth and can offer a fast evaluation of the analytic gradients. The root-mean-square (rms) error of the global fit is 0.42 kcal mol^{-1} for energy points ranging from the global minimum up to 15

kcal mol^{-1} above the C(^1D)+D_2 asymptote, and the maximum error is 2.79 kcal mol^{-1}, which is in a region far from the reaction path. It should be noted that the fitting errors in dynamically important regions are much lower, because energy points in these regions were given very high weights in the fitting process, which improves the quality of our PES.

B. Quantum dynamics calculations

The quantum dynamics (QD) calculations for the C(^1D)+D_2(ν=0,j=0)→CD+D reaction were performed using the DIFFREALWAVE code [13, 14]. The propagation of the wave packet was carried out in terms of product Jacobi coordinates R', r' and γ' (see Supplementary Fig. 1b). The final wave packet was analysed to extract the state-to-state scattering matrix (S-matrix) elements for the lowest 3 vibrational states and the lowest 25 rotational states of the product CD for all the total angular momenta (Js) involved in this work. All parameters used in the calculations are given in Supplementary Table 3.

Numerous test calculations were performed to check the convergence of all the parameters, especially for the grid sizes, center of initial wave packet (R_0), and analysis line (R'_∞). We find it necessary to extend the grids to rather large values. The initial wave packet should be centered in the asymptotic reactant channel in order to include the long-range interactions with which we are most concerned in this work. In addition, in order to obtain accurate state-to-state dynamical quantities, the R'_∞ must be placed in the product asymptotic region of the surface where the potential is as flat as possible. It is relatively easy to meet the above criteria for small Js. However, it is imperative to choose larger values of R_0 and R'_∞ because the centrifugal potential falls off slower as the J increases. Furthermore, it should be noted that, at collision energies above 0.3 eV, the QD integral cross sections calculated on the ZMB-a surface are not fully converged, but the variations are expected to be very small, and thus they are also included in Fig. 2 for comparison.

V. SUPPLEMENTARY REFERENCES

[1] Sato, K., Ishida, N., Kurakata, T., Iwasaki, A. & Tsunashima, S. Reactions of $C(^1D)$ with H, HD and D_2: Kinetic isotope effect and the CD/CH branching ratio. *Chem. Phys.* **237,** 195–204 (1998).

[2] Werner, H.-J. & Knowles, P. J. An efficient internally contracted multiconfiguration-reference configuration interaction method. *J. Chem. Phys.* **89,** 5803–5814 (1988).

[3] Knowles, P. J. & Werner, H.-J. An efficient method for the evaluation of coupling coefficients in configuration interaction calculations. *Chem. Phys. Lett.* **145,** 514–522 (1988).

[4] Werner, H.-J. & Knowles, P. J. A second order multiconfiguration SCF procedure with optimum convergence. *J. Chem. Phys.* **82,** 5053–5063 (1985).

[5] Knowles, P. J. & Werner, H.-J. An efficient second-order MC SCF method for long configuration expansions. *Chem. Phys. Lett.* **115,** 259–267 (1985).

[6] Dunning, T. H. Gaussian basis sets for use in correlated molecular calculations. I. The atoms boron through neon and hydrogen. *J. Chem. Phys.* **90,** 1007–1023 (1989).

[7] Kendall, R. A., Dunning, T. H. & Harrison, R. J. Electron affinities of the first-row atoms revisited. Systematic basis sets and wave functions. *J. Chem. Phys.* **96,** 6796–6806 (1992).

[8] Werner, H.-J. *et al.* (2006). MOLPRO, Version 2006.1, a package of *ab initio* programs, see http://www.molpro.net.

[9] Bussery-Honvault, B., Julien, J., Honvault, P. & Launay, J.-M. Global $1^1A''$ potential energy surface of CH_2 and quantum dynamics of a sideways insertion mechanism for the $C(^1D)+H_2 \rightarrow CH(^2\Pi)+H$ reaction. *Phys. Chem. Chem. Phys.* **7,** 1476–1481 (2005).

[10] Herzberg, G. & Johns, J. W. C. Spectrum and structure of singlet CH_2. *Proc. R. Soc. London, Ser. A* **295,** 107–128 (1966).

[11] Irikura, K. K. Experimental vibrational zero-point energies: Diatomic molecules. *J. Phys. Chem. Ref. Data* **36,** 389–397 (2007).

[12] Ruscic, B. *et al.* IUPAC critical evaluation of thermochemical properties of selected radicals. Part I. *J. Phys. Chem. Ref. Data* **34,** 573–656 (2005).

[13] Hankel, M., Smith, S. C., Allan, R. J., Gray, S. K. & Balint-Kurti, G. G. State-to-state reactive differential cross sections for the $H+H_2 \rightarrow H_2+H$ reaction on five different potential energy surfaces employing a new quantum wavepacket computer code: DIFFREALWAVE. *J. Chem. Phys.* **125,** 164303 (2006).

[14] Hankel, M., Smith, S. C., Gray, S. K. & Balint-Kurti, G. G. DIFFREALWAVE: A parallel real wavepacket code for the quantum mechanical calculation of reactive state-to-state differential cross sections in atom plus diatom collisions. *Comput. Phys. Commun.* **179,** 569–578 (2008).
